# Identifying EGFR mutation-induced drug resistance based on alpha shape model analysis of the dynamics

**DOI:** 10.1186/s12953-016-0102-0

**Published:** 2016-09-08

**Authors:** Lichun Ma, Bin Zou, Hong Yan

**Affiliations:** Department of Electronic Engineering, City University of Hong Kong, 83 Tat Chee Avenue, Kowloon, Hong Kong

**Keywords:** Epidermal growth factor receptor (EGFR), Non-small-cell lung cancer (NSCLC), Gefitinib, Drug binding site, Molecular dynamics, Alpha shape modeling, Solid angle

## Abstract

**Background:**

Epidermal growth factor receptor (EGFR) mutation-induced drug resistance is a difficult problem in lung cancer treatment. Studying the molecular mechanisms of drug resistance can help to develop corresponding treatment strategies and benefit new drug design.

**Methods:**

In this study, Rosetta was employed to model the EGFR mutant structures. Then Amber was carried out to conduct molecular dynamics (MD) simulation. Afterwards, we used Computational Geometry Algorithms Library (CGAL) to compute the alpha shape model of the mutants.

**Results:**

We analyzed the EGFR mutation-induced drug resistance based on the motion trajectories obtained from MD simulation. We computed alpha shape model of all the trajectory frames for each mutation type. Solid angle was used to characterize the curvature of the atoms at the drug binding site. We measured the knob level of the drug binding pocket of each mutant from two ways and analyzed its relationship with the drug response level. Results show that 90 % of the mutants can be grouped correctly by setting a certain knob level threshold.

**Conclusions:**

There is a strong correlation between the geometric properties of the drug binding pocket of the EGFR mutants and the corresponding drug responses, which can be used to predict the response of a new EGFR mutant to a drug molecule.

## Background

Lung cancer is the leading cause of cancer deaths worldwide [1–3]. Non-small-cell lung carcinoma (NSCLC) is the most common lung cancer type, accounting for over 80 % of all the lung cancer cases [4, 5]. Epidermal growth factor receptor (EGFR) is found to overexpress in about 60 % of NSCLC patients, making it a target of many treatment strategies [6–8]. EGFR, also called HER1 or ErbB-1, is a member of the ErbB family, which also contains HER2 (ErbB-2), HER3 (ErbB-3) and HER4 (ErbB-4) [9, 10]. It is a functional protein that can be activated through binding with cognate ligands, such as epidermal growth factor [11]. On ligand binding, EGFR can form a homodimer (EGFR-EGFR) or heterodimers (dimerization with other family members), leading to the phosphorylation of specific residues at the intracellular tyrosine kinase (TK) domain. These phosphorylated residues act as docking sites for downstream proteins, triggering the downstream pathways that modulate cellular proliferation and survival [12, 13]. However, aberrant EGFR signaling, sometimes caused by mutations at the EGFR TK domain, can lead to tumor growth and progression in the lung [14–16]. Gefitinib is a commonly used drug to target the EGFR TK domain to block the activation of EGFR and its downstream signaling [17–22]. Nevertheless, after a period of treatment, drug resistance occurs usually due to a second mutation (e.g. T790M) or the activating of other receptor tyrosine kinases (e.g. c-Met) [23–26].

A lot of research has focused on drug resistance mechanisms in lung cancer [27–30]. Computational methods are successfully applied in these studies, benefiting from their advantages of low cost, easy implementation and capacity of processing large datasets [31–33]. Zhu et al. employed support vector machine based classifiers to correlate the collected features (clinicopathologic features and immunomarkers) and the overall survival of NSCLC patients [34]. Wang et al. used the personal features of 168 NSCLC patients coupled with EGFR mutant-drug binding free energy features to build a classification model to predict the drug response levels, and obtained a best testing accuracy of 95.83 % [35].

To decode the EGFR mutation-induced drug resistance, it is very important to analyze the interaction between the EGFR mutants and a drug molecule. The geometric properties of the drug binding pocket can affect the binding affinity of two molecules. Intuitively, a concave shape may have a higher binding affinity with a drug molecule than a convex shape because of structural complementary characteristics. In addition, a shape with a low convex degree could bind more tightly on a drug molecule relative to a shape with high convex degree. Therefore, different from other studies which focused on personal features, energy features as well as immunomarkers, we use the geometric properties of the drug binding pocket of the EGFR mutants to identify the drug resistance mechanisms. Compared with the EGFR-drug binding free energy used by Wang et al. [35], which shows the overall binding affinity, the geometric features can provide specific structural information of each atom at the drug binding pocket. These structural information offers clues on how to modify the drug structure and the spatial relations of its atoms with EGFR to overcome the resistance problem.

In this work, we study the EGFR mutation-induced drug resistance by analyzing structural properties of the mutants in a dynamic form, based on the motion trajectories obtained from molecular dynamics (MD) simulation. The clinical data of EGFR mutation type and the drug response of patients were collected from Queen Mary Hospital in Hong Kong. We employed Rosetta to model the EGFR mutants based on the crystal structure of wild-type (WT) EGFR and the mutant sequences [36]. Then Amber [37] was used to conduct MD simulation to show the dynamic evolution of the EGFR mutant-drug system. We extracted the trajectory frames of each mutant and computed the alpha shape model [38, 39] of each frame, in order to describe the mutant with geometric models. Solid angle [33, 40] was evaluated to characterize the curvature properties of the atoms at the drug binding site. Finally, we computed the knob level of the drug binding pocket of each mutant and analyzed its relationship with the drug response level. Results show that 90 % of the mutants can be grouped correctly by setting a certain knob level threshold.

## Methods

### Clinical data and ethics statement

The clinical data of NSCLC patients were collected from Queen Mary Hospital in Hong Kong. This study was approved by Institutional Review Board of the University of Hong Kong/Hospital Authority Hong Kong West Cluster. Specifically, there are 137 patients belonging to 30 EGFR mutation types. The mutation type of each patient was obtained through an EGFR mutation test, in which the mutations of the EGFR gene were detected by analyzing the DNA of the tumor sample. All the patients in our dataset took the tyrosine kinase inhibitor gefitinib in the treatments. The potency of the drug was measured by drug response level (*RL*), which was obtained by evaluating the selected target lesions of the patients, based on measurement methods such as computed tomography (CT) scan, magnetic resonance imaging (MRI) and chest X-ray. According to the changes of the target lesions, the *RL* can be categorized into four levels, *complete response* (*RL* = 1), *partial response* (*RL* = 2), *stable disease* (*RL* = 3) and *progressive disease* (*RL* = 4). *Complete response* indicates that all the target lesions have disappeared. *Partial response* means that at least a 30 % decrease of the target lesions is measured compared with the sum of the longest diameters (LDs) of the target lesions before treatment. *Stable disease* and *progressive disease* take the smallest sum of the LDs as reference. *Stable disease* donates that no sufficient decrease or increase is noted, while at least a 20 % increase is measured in *progressive disease*. For simplicity, we can combine the *complete response* and *partial response* to a drug *Response* group, while the other two are categorized into a *No-response* group. Table 1 shows the *RL* of each mutant, derived by the median *RL* value of the patients harboring this EGFR mutation type. These EGFR mutations as well as their corresponding *RL*s were used for further analysis in our study.

**Table 1:** Drug response of the mutants

Mutant	*RL*	Mutant	*RL*
delE746_T751insV	1	R776HL858R	2
delE746_T751insVA	1	delE746_T751insA	3
delE746_A750	2	delL747_A750insP	3
delE746_A750insAP	2	dulS768_D770	3
delE746_S752insV	2	G719CS768I	3
delE746_T751insI	2	R831H	3
delL747_A755insSKG	2	S768IV774M	3
delL747_P753insS	2	delE709_T710insD	4
delL747_T751	2	delL747_K754insANKG	4
delT751_I759insN	2	dulH773	4
E709KL858R	2	dulN771_H773	4
G719AL858R	2	E709AG719A	4
G719AL861Q	2	G724SL861Q	4
L858R	2	K757R	4
L861Q	2	L861R	4

### Modeling of EGFR mutants

**Fig. 1:**
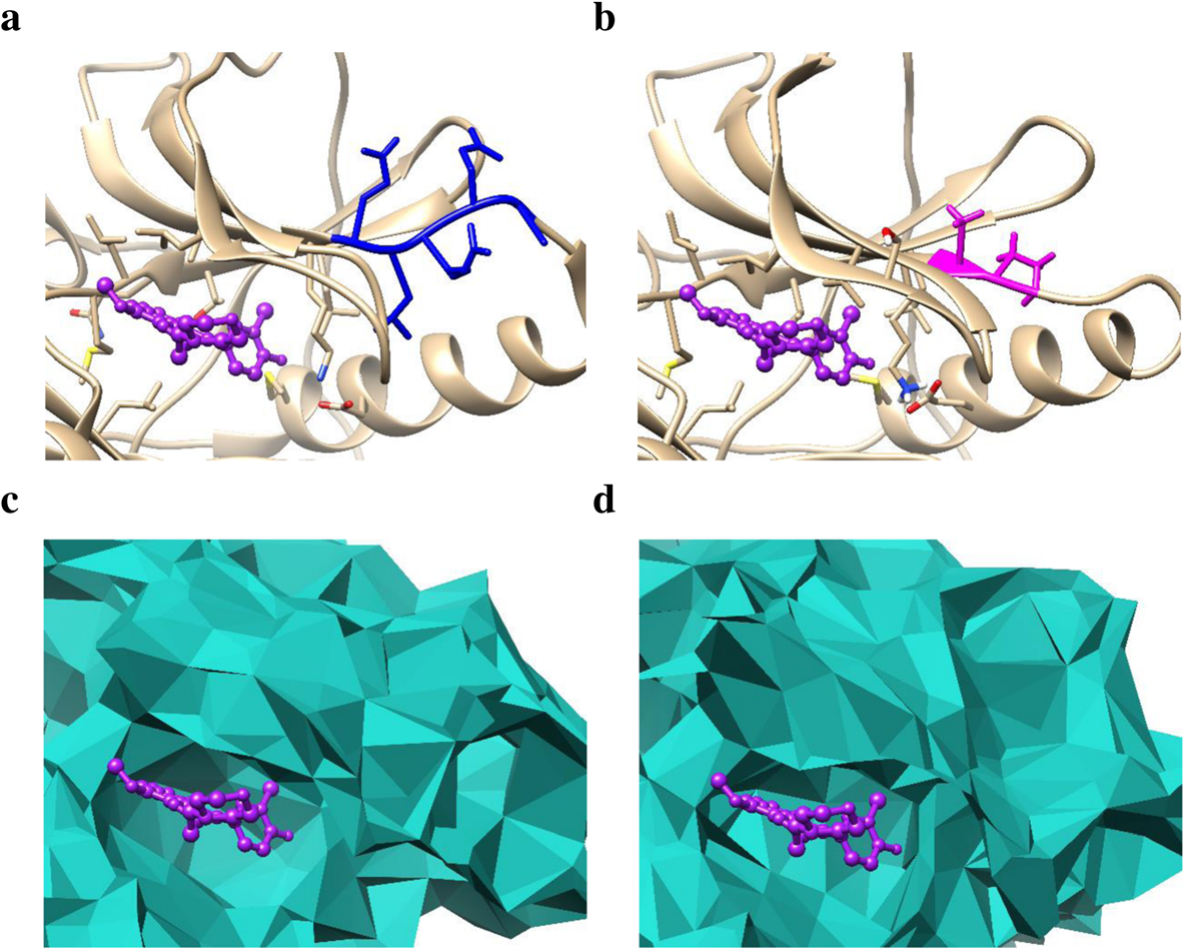
**a** and **b** show the comparison of the crystal structures of WT EGFR and the mutant delE746_A750insAP. **c** and **d** are the alpha shape models of the drug binding pocket of (**a**) and (**b**), respectively. The original site is colored blue while the corresponding mutant site is shown in magenta. The drug molecule (gefitinib) is displayed in purple

Only several EGFR mutant crystal structures are available from the Protein Data Bank (PDB) [41], due to the cost and complexity in structure determination by experiments. We adopted the released mutant structures from PDB, such as L858R (PDB: 2ITZ), while modeled most of them using Rosetta [36]. We carried out Rosetta high-resolution *ddg_monomer* (HRDM) protocol and *comparative modeling* (CM) protocol to generate the EGFR mutants based on the crystal structure of WT EGFR (PDB: 2ITY) [42, 43]. Rosetta *ddg_monomer* was applied to predict the point mutation, such as L861Q and G719C_S768I. Other mutation types such as delE746_A750 (deletion), dulN771_H773 (duplication) and delE746_A750insAP (modification) were modeled by using the CM protocol. We further refined the predicted mutant structures using Amber [37], where 1000 steps of minimization were conducted to optimize the structures. Figures 1a and b show the comparison of the WT EGFR and the mutant delE746_A750insAP modeled using Rosetta.

### Molecular dynamics (MD) simulation

MD simulation can be used to study the interaction of molecules for a fixed period of time, and record the movements as trajectories which are determined by solving Newton’s equations of motion. We employed Amber 12 [37] to conduct MD simulation to the EGFR mutant-drug complex. The complex was obtained by aligning the predicted mutant structure to the WT EGFR and appending drug molecule to it. Amber *ff99SB* force field is adopted to describe the forces between particles in the simulation. As the simulation was performed in a solvent environment, we solvated the complex into a periodic TIP3P water box, where molecules exit one side of the box will wrap to the other side. Then we conducted a series of steps (minimization, heating, density equilibration, and constant pressure equilibration) to equilibrate the system. At the environment of desired temperature, density and pressure, the production MD simulation was performed for 2 ns. We saved the motion trajectories every 10 ps and a total of 200 trajectory frames were collected for each mutant-drug complex.

### Alpha shape modeling and solid angle calculation

After the motion trajectories of each mutant were obtained, we carried out alpha shape modeling for each trajectory frame, in order to show the surface geometric properties of the mutant structure. The alpha shape [38, 39] can provide effective approximation of the original shape of a molecule with a computational geometric model. We employed Computational Geometry Algorithms Library (CGAL) [44] to generate the weighted alpha shape models of the EGFR mutants. Figures 1c and d show the alpha shape models of the drug binding pocket of WT EGFR and the mutant delE746_A750insAP, respectively.

Then we used solid angle to characterize the geometric properties of each atom at the drug binding site. Solid angle describes the curvature by providing a value (in the range of [−1, 1]) to show the concave or convex properties of each surface atom. If the solid angle value falls in [−1, 0), the shape is defined as a concave one while a convex shape is obtained if the value is in (0, 1]. The detailed definition of solid angle can be found in [33, 40].

## Results and Discussion

Based on the motion trajectories obtained from MD simulation, we carried out alpha shape modeling for all the 30 EGFR mutants. A total of 200 trajectory frames were collected in the production MD simulation process. Thus, the alpha shape model was built for 200 times for each mutant. Then we used solid angle to characterize the atoms at the drug binding site. In this study, the drug binding site of a mutant structure was defined as the amino acid residues at the drug binding site of the WT EGFR. Specifically, a total of 14 residues of 102 atoms are involved at the drug binding site of the WT EGFR. A few mutations locate just at the drug binding site, such as G719A_L858R, while the majority of mutations do not.

**Fig. 2:**
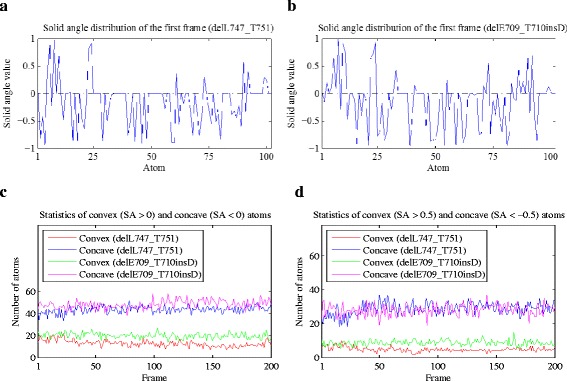
**a** and **b** show the solid angle values at the drug binding site of the first trajectory frame of the mutants delE709_T710insD and delL747_T751, respectively. The dark dashed lines indicate the position of 0. **c** and **d** demonstrate the number of convex and concave atoms with different solid angle value thresholds at the drug binding site of the two mutants

We computed the solid angles of all the atoms at the drug binding site, and the values of those who were not at the surface of the drug binding site were set to zero. Figures 2a and b show the solid angle values at the first trajectory frame of the mutants delL747_T751 (*RL* = 2) and delE709_T710insD (*RL* = 4). According to the *RLs* of the two mutants, they are classified to the drug *Response* and *No-response* group, respectively. As shown in the two figures, the number of convex atoms of the mutant delE709_T710insD is more than that of delL747_T751. Then we counted the number of convex and concave atoms for all the 200 trajectory frames of the two mutants respectively, and even made a further step to calculate the number of atoms with SA (solid angle value) > 0.5 or SA < − 0.5. Figures 2c and d indicate that the concave indexes of the two mutants are mixed together while the convex ones are clearly separated. Consequently, we only consider the convex-related characteristics of the mutants in the following studies.

**Fig. 3:**
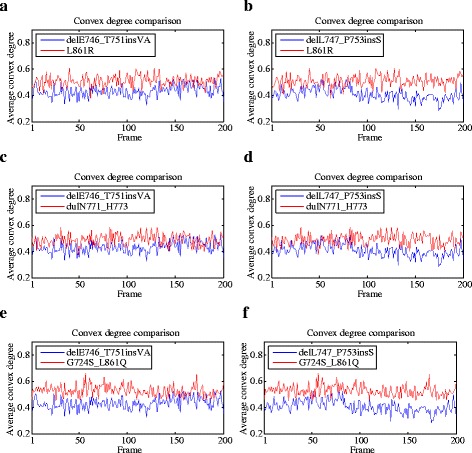
The comparison of average convex degrees of the drug *Response* mutants delE746_T751insVA (*RL* = 1, shown in **a**, **c** and **e**) and delL747_P753insS (*RL* = 2, shown in **b**, **d** and **f**), and the *No-response* ones L861R (*RL* = 4), dulN771_H773 (*RL* = 4) and G724S_L861Q (*RL* = 4). The drug *Response* mutants are shown in blue, while the *No-response* ones are colored red

First, we computed the average convex degree of the drug binding site for all the motion trajectory frames of each mutant. For a specific trajectory frame, the average convex degree was calculated as the sum of all the solid angle values of convex atoms at the drug binding site divided by the total number of convex atoms in this area. Figure 3 shows the comparison of average convex degree of the drug *Response* mutants delE746_T751insVA (*RL* = 1) and delL747_P753insS (*RL* = 2), and the *No-response* ones L861R (*RL* = 4), dulN771_H773 (*RL* = 4) and G724S_L861Q (*RL* = 4). Although the two groups of mutants cannot be separated perfectly, the average convex degree of delE746_T751insVA and delL747_P753insS are generally lower than that of L861R, dulN771_H773 and G724S_L861Q.

**Fig. 4:**
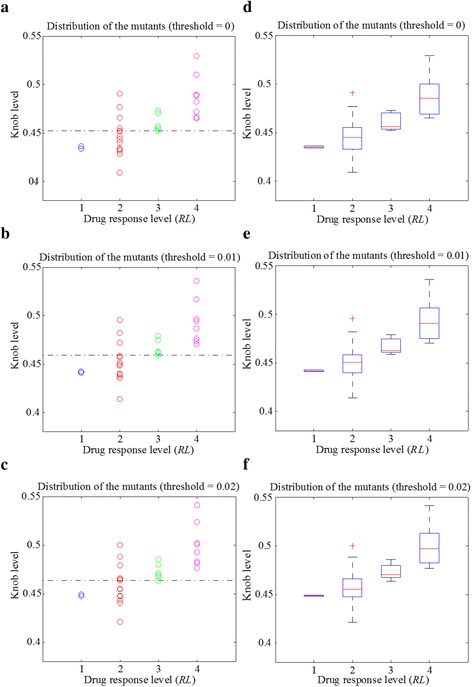
The relationship of knob level and drug response level of the 30 mutants, with the solid angle value threshold setting to 0, 0.01 and 0.02, respectively. **a** to **c** show the scatter plots of the mutants for each drug response level. **d** to **f** are the corresponding box plots of (**a**) to (**c**)

Then we defined knob level, the mean of the average convex degrees for the 200 trajectory frames, to characterize the drug binding site of each mutant. The solid angle values of some atoms are very close to zero (Figs. 2a and b), making a large difference on the average convex degree of each trajectory frame. Therefore, we set a solid angle value threshold to avoid their influence. Afterwards, we explored the relationship between knob level and drug response level of the 30 mutants. The results are shown in Fig. 4, with solid angle value threshold setting to 0, 0.01 and 0.02 respectively. Figures 4a to c are the scatter plots of the mutants. When the threshold equals to 0.01, the mutants of the *Response* and *No-response* groups can be separated with only 3 errors (3 mutants belonging to *RL* = 2 are wrongly categorized). Figures 4d to f shows the corresponding box plots of Figs. 4a to c, respectively. As shown in these figures, the median knob level values (bands inside the boxes) of the four drug response level groups are clearly separated. In addition, the main bodies (boxes) of the two groups (*Response* and *No-response*) of mutants are at different knob levels, when the threshold equals to 0.01 or 0.02.

**Fig. 5:**
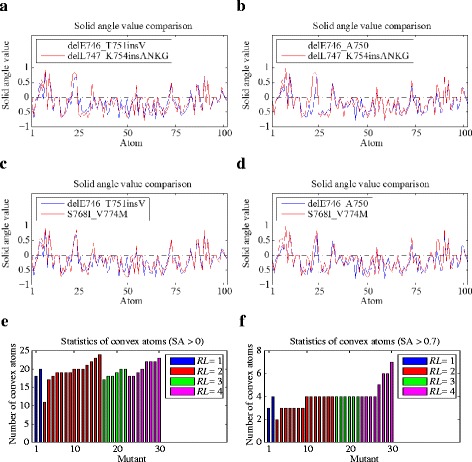
**a** to **d** presents the comparison of the solid angle values of the atoms at the average binding site of the drug *Response* mutants delE746_T751insV (*RL* = 1, **a﻿** and **c**) and delE746_A750 (*RL* = 2, **b** ﻿and **d**), and the *No-response* ones delL747_K754insANKG (*RL* = 4) and S768I_V774M (*RL* = 3). The drug *Response* and *No-response* mutants are shown in blue and red, respectively. **e** and **f** show the statistics of the convex atoms and the atoms with SA > 0.7 at the average binding site of the 30 mutants. The mutants of each response level group are sorted in an ascending order by the number of convex atoms. Blue, red, green and magenta correspond to mutants with *RL *= 1, 2, 3 and 4

Besides the aforementioned definition of knob level, we alternatively computed the mean of the solid angle value of each atom at the drug binding site from the 200 trajectory frames. In this way, an average drug binding site of each mutant was obtained. Thus, we used the average convex degree of this drug binding site as the knob level to describe the mutant. Figures 5a to d show the comparison of the solid angle values of the atoms at the average binding site of the drug *Response* mutants delE746_T751insV (*RL* = 1) and delE746_A750 (*RL* = 2), and the *No-response* ones delL747_K754insANKG (*RL* = 4) and S768I_V774M (*RL* = 3). Although there is not much difference between the total number of convex atoms of the *Response* and *No-response* groups, the solid angle values of convex atoms in the *No-response* group are generally greater than that of the *Response* group. We also counted the number of convex atoms (SA > 0) and the number of atoms with SA > 0.7 at the average drug binding site of each mutant (Figs. 5e and f). The number of convex atoms of the 30 mutants is irregular for different response level groups. However, the number of atoms with SA > 0.7 of the *No-response* group is equal to or more than that of the *Response* group.

**Fig. 6:**
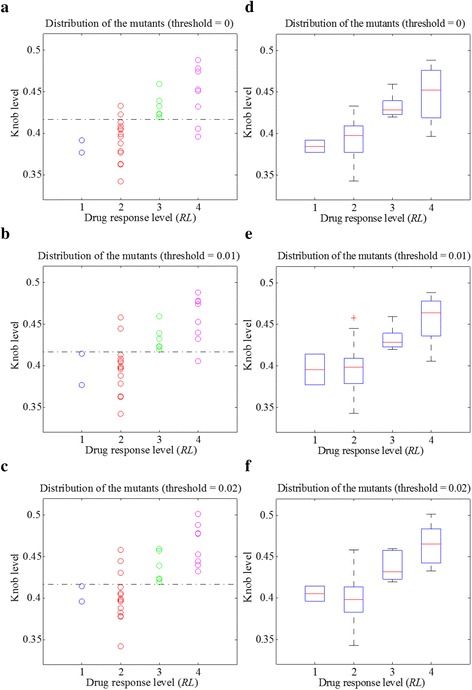
The relationship between the knob level of the average binding site and the drug response level of the mutants, with the solid angle value threshold setting to 0, 0.01 and 0.02 respectively

Afterwards, we used the average convex degree (the sum of all the solid angle values of convex atoms at the average drug binding site divided by the total number of convex atoms in this area) to characterize the knob level of the average drug binding site of each mutant. Similarly, some solid angle values are close to zero, and we set a solid angle value threshold to remove their influence. The relationship between knob level and drug response level of the mutants are shown in Fig. 6. When the solid angle value threshold equals to 0.01 or 0.02, only three mutants of the two groups (*Response* and *No-response* groups) are wrongly distinguished by setting a certain knob level boundary (dark dashed lines in Figs. 6b and c), showing an accuracy of 90 % (27/30). In addition, the main bodies of the two groups of mutants are in different knob level ranges.

## Conclusions

In this study, we analyzed the motion trajectories of the EGFR mutants obtained from MD simulation. The EGFR mutant structures were generated using Rosetta. Amber was employed to carry out MD simulation of the EGFR mutant-drug system. The motion trajectories were collected every 10 ps and a total of 200 frames were obtained for each mutant. Then we computed alpha shape model of each trajectory frame and characterized the curvature of the atoms at the drug binding site using the solid angle. In one aspect, we calculated the average convex degree at the drug binding site of each trajectory frame, and obtained the knob level of a mutant by computing the mean value of the convex degrees of the 200 frames. On the other hand, we calculated the mean of the solid angle value of each atom at the drug binding site from the 200 trajectory frames. In this way, an average drug binding site could be obtained, and the average convex degree of this binding site was calculated to measure the knob level of a mutant. Finally, we analyzed the relationship between the knob level of the drug binding site and the drug response level of the mutants. Results show that 90 % of the mutants can be grouped correctly by setting a certain knob level threshold.

To validate our model, we can compare the predicted response with the clinically obtained drug response of the EGFR mutants collected from literature. Meanwhile, the newly collected clinical data provide resources to refine our model to make further prediction more accurately. For example, L858R_T790M is a well-known mutant which is resistant to gefitinib. Then we can compute its alpha shape model and derive the knob level of the drug binding pocket in the aforementioned two ways. As the best grouping accuracy is achieved when the solid angle threshold equals to 0.01 for both the two way situations (Figs. 4b and b). We set the threshold to 0.01, and obtained the knob level of 0.5298 and 0.4964 with the two approaches, respectively. According to knob level thresholds of the drug *Response* mutants and the *No-response* ones in Figs. 4b and b, the mutant of L858R_T790M can be categorized to the *No-response* group, which is consistent with our knowledge.

By using the obtained relationships between knob level and drug response level of the EGFR mutants, we can have a general classification of a new EGFR mutant before measuring the drug response from clinical evaluation. We can model the 3D structure as well as the alpha shape of a new EGFR mutant to derive the knob level of the drug binding pocket. According to the obtained knob level threshold of the drug *Response* and *No-response* groups, we are able to predict the drug response of the new mutant. The results can be used for clinical guidance and can benefit the patients with a more effective therapy.
